# Some Open Questions About the Anisotropic Random Walks

**DOI:** 10.3390/e26121082

**Published:** 2024-12-11

**Authors:** Endre Csáki, Antónia Földes

**Affiliations:** 1 Alfréd Rényi Institute of Mathematics, P.O. Box 127, H-1364 Budapest, Hungary; csaki.endre@renyi.hu; 2 Department of Mathematics, College of Staten Island, The City University of New York, 2800 Victory Blvd., Staten Island, NY 10314, USA

**Keywords:** anisotropic random walk, strong approximation, two-dimensional Wiener process, local time, 60F17, 60G50, 60J65, 60F15, 60J10

## Abstract

Between 2007 and 2018, we collaborated extensively with Pál Révész and Miklós Csörgő on many of the problems discussed in this paper. Over the past six years, we have continued to explore these issues, and here, we present some of the most intriguing open questions in these areas. This paper compiles key results from a dozen of our previous works, providing the necessary background to frame these compelling unresolved questions.

## 1. Introduction

The investigation of the two-dimensional anisotropic random walk goes back almost fifty years. The first papers were written by Silver, Shuler and Lindenberg [1]; Shuler [2]; Seshadri, Lindenberg and Shuler [3] and Westcott [4] followed by Heyde [5,6]; Roerdink and Shuler [7]; and den Hollander [8]. Let us begin with the definition. We consider a symmetric nearest-neighbor random walk on $Z^{2},$ such that its transition probabilities only depend on the vertical coordinate. To be precise, here is the formal definition: the anisotropic random walk $\left\{C\left(N\right)=\left(C_{1}\left(N\right),C_{2}\left(N\right)\right),\;\;N=0,1,2,\ldots \right\}$ on $Z^{2}$ has the following transition probabilities:

(1) $$\begin{array}{ccc} P(C(N+1) & = & (k+1,j)|C(N)=(k,j))=\\ P(C(N+1) & = & \left(k-1,j\right)|C\left(N\right)=\left(k,j\right))=\frac{1}{2}-p_{j},\\ P(C(N+1) & = & (k,j+1)|C(N)=(k,j))=\\ P(C(N+1) & = & \left(k,j-1\right)|C\left(N\right)=\left(k,j\right))=p_{j}, \end{array}$$

for $\left(k,j\right)\in Z^{2}$, N=0,1,2,… We will suppose that $0<p_{j}\leq 1/2$ and $\min_{j\in Z}p_{j}<1/2$ and that C(0)=(0,0).

Obviously, this model contains the simple symmetric walk, when $p_{i}=1/4$ for all i=0,±1,±2,… The simple symmetric walk has been discussed extensively, so we just mention a few important works here: Erdős and Taylor [9], Dvoretzky and Erdős [10] and Révész [11]. The condition $\min_{j\in Z}p_{j}<1/2$ excludes the case where the walk is one-dimensional. However, the case when all $p_{i}=1/2$ except one is a particularly famous case. More precisely, if $p_{0}=1/4$ and all the other $p_{i}=1/2$, we have the **two-dimensional comb** model, which has been investigated by Weiss and Havlin [12]; Bertacchi [13]; Bertacchi and Zucca [14]; and us [15,16]. We will mention some other special cases later on.

## 2. Recurrence and Transience

One of the most important issues is whether a particular random walk is transient or recurrent. We investigated this question for the anisotropic random walk in [17]. It turned out that to obtain criteria for recurrence, only the simple application of the famous Nash–Williams theorem is required [18]. For completeness, we recall this theorem here. To do so, we need some notations and definitions. Let (X,Y,p) be a Markov chain with a countable state space X and a process Y with transition probabilities p(u,v). The chain is called *reversible* if there exist strictly positive weights $\pi_{u}$ for all u∈X such that

(2) $$\pi_{u}p\left(u,v\right)=\pi_{v}p\left(v,u\right).$$

For reversible chains, it is convenient to introduce the notation

$$a\left(u,v\right):=\pi_{u}p\left(u,v\right).$$

Our anisotropic walk introduced above is a Markov chain on the state space $X=Z^{2}$, with the transition probabilities defined in (1), and it is reversible with the strictly positive weights

$$\pi \left(k,j\right)=\frac{1}{p_{j}},$$

with $p_{j},j=0,\pm 1,\pm 2,\ldots$ defined in (1). Thus, we have, for neighboring sites,

(3) $$\begin{array}{ccc} a\left((k,j),(k,j+1)\right) & = & a\left((k,j),(k,j-1)\right)=1\\ a\left((k,j),(k+1,j)\right) & = & a\left((k,j),(k-1,j)\right)=\frac{1}{2p_{j}}-1 \end{array}$$

(and for non-nearest-neighbor sites a(·,·)=0). Our Markov chain is also time-homogeneous and irreducible (it is possible to get to any state from any state with positive probability). The invariant measure is defined by

$$\mu \left(u\right)=\sum_{v}\mu \left(v\right)p\left(v,u\right),$$

where the summation goes over the four nearest neighbors of u. For u=(k,j), we obtain

(4) $$\mu \left(u\right)=\mu \left(k,j\right)=\pi \left(k,j\right)=\frac{1}{p_{j}},\;\left(k,j\right)\in Z^{2}.$$

Now we recall the Nash–Williams theorem:

**Theorem** **1** ([18]). *Suppose that (X,Y,p) is a reversible Markov chain and that $X={⋃}_{k=0}^{\infty }\Lambda^{k}$ where $\Lambda^{k}$ are disjoint. Suppose further that $u\in \Lambda^{k}$ and a(u,v)>0 together imply that $v\in \Lambda^{k-1}⋃\Lambda^{k}⋃\Lambda^{k+1},$ and that for each k, the sum $\sum_{u\in \Lambda^{k},v\in X}a\left(u,v\right)<\infty .$ Let $\left[\Lambda^{k},\Lambda^{k+1}\right]$ denote the set of pairs (u,v) such that $u\in \Lambda^{k}$ and $v\in \Lambda^{k+1}.$ The Markov chain is recurrent if*

(5) $$\sum_{k=0}^{\infty }{\left(\sum_{(u,v)\in \left[\Lambda^{k},\;\Lambda^{k+1}\right]}a\left(u,v\right)\right)}^{-1}=\infty .$$

To apply this theorem, let $\Lambda^{k}$ be the set of the 8k lattice points on a square of width 2k, centered at the origin. Furthermore, let $\left[\Lambda^{k},\;\Lambda^{k+1}\right]$ be the set of the 8k+4 nearest-neighbor pairs (edges) between $\Lambda^{k}$ and $\Lambda^{k+1}.$

It is easy to see from (3) that the sum in (5) is equal to

$$\sum_{k=0}^{\infty }{\left(2\left(\sum_{j=-k}^{k}\left(\frac{1}{2p_{j}}-1\right)+\sum_{j=-k}^{k}1\right)\right)}^{-1}=\sum_{k=0}^{\infty }{\left(\sum_{j=-k}^{k}\frac{1}{p_{j}}\right)}^{-1}.$$

Consequently, we have

**Theorem** **2.** *The anisotropic walk is recurrent if*

(6) $$\sum_{k=0}^{\infty }{\left(\sum_{j=-k}^{k}\frac{1}{p_{j}}\right)}^{-1}=\infty .$$

This implies the following.

**Corollary** **1.** *If $\min_{j\in Z}p_{j}>0$, then the anisotropic walk is recurrent.*

Of course, it is very tempting to believe that the converse of this statement is true as well. Unfortunately, *we cannot prove* that if

(7) $$\sum_{k=0}^{\infty }{\left(\sum_{j=-k}^{k}\frac{1}{p_{j}}\right)}^{-1}<\infty ,$$

then the anisotropic walk is transient, but we managed to show a somewhat weaker result:

**Theorem** **3** ([17]). *Assume that*

(8) $$\sum_{j=-k}^{k}\frac{1}{p_{j}}=Ck^{1+A}+O\left(k^{1+A-\delta }\right),\;k\to \infty$$

*for some C>0, A>0 and 0<δ≤1. Then, the anisotropic random walk is transient.*

So, our first open question is

**Question** **1.** *Find an if and only if criterion for the recurrence of the anisotropic walk.*

## 3. Strong Approximation

Let us start with some history. After the early works given in [1,2], Heyde [5] proved an almost sure approximation for $C_{2}\left(N\right),$ the second coordinate of the anisotropic walk, under the following conditions:

(9) $$n^{-1}\sum_{j=1}^{n}p_{j}^{-1}=2\gamma +o\left(n^{-\eta }\right),\;n^{-1}\sum_{j=1}^{n}p_{-j}^{-1}=2\gamma +o\left(n^{-\eta }\right)$$

as n→∞ for some constants γ, 1<γ<∞ and 1/2<η<∞.

In our paper [19], we proved for {C(N),N=0,1,2,…} the following joint strong approximation theorem:

**Theorem** **4.** *Under condition* (9)*, with 1/2<η≤1 on an appropriate probability space for the random walk $\left\{C\left(N\right)=\left(C_{1}\left(N\right),C_{2}\left(N\right)\right),\;\;N=0,1,2,\ldots \right\}$, one can construct two independent standard Wiener processes $\left\{W_{1}\left(t\right);\;t\geq 0\right\}$, $\left\{W_{2}\left(t\right);\;t\geq 0\right\}$ so that, as N→∞, we have, with any ε>0,*

$$\left|C_{1}\left(N\right)-W_{1}\left(\frac{\gamma -1}{\gamma }\;N\right)\right|+\left|C_{2}\left(N\right)-W_{2}\left(\frac{1}{\gamma }\;N\right)\right|=O\left(N^{5/8-\eta /4+\varepsilon }\right)\;a.s.$$

We also considered a special case of this result which dealt with the so-called periodic case. The anisotropic walk is called **periodic** if $p_{i}=p_{i+L}$ for each i∈Z with some positive integer L≥1. By indicating this periodicity with the superscript *P*, we obtained a somewhat better approximation, as the next theorem shows.

- **Periodic walk**

**Theorem** **5.** *On an appropriate probability space for the periodic anisotropic random walk,*

$$\{C^{P}\left(N\right)=\left(C_{1}^{P}\left(N\right),C_{2}^{P}\left(N\right)\right);\;N=0,1,2,\ldots \},$$

*one can construct two independent standard Wiener processes, $\left\{W_{1}\left(t\right);\;t\geq 0\right\}$ and $\left\{W_{2}\left(t\right);\;t\geq 0\right\}$, so that, as N→∞, we have, with any ε>0,*

(10) $$\left|C_{1}^{P}\left(N\right)-W_{1}\left(\frac{\gamma -1}{\gamma }\;N\right)\right|+\left|C_{2}^{P}\left(N\right)-W_{2}\left(\frac{1}{\gamma }\;N\right)\right|=O\left(N^{1/4+\varepsilon }\right)\;a.s.,$$

*where*

(11) $$\gamma =\frac{\sum_{j=1}^{L}p_{j}^{-1}}{2L}.$$

**Remark** **1.** *In what follows, we will talk about the different important classes of anisotropic random walks, like the two-dimensional comb anisotropic random walk, the periodic anisotropic random walk and many others. To simplify the language from now on, we will often call these the (two-dimensional) comb walk and periodic walk, thus avoiding the use of anisotropic random expression.*

It is a natural question to ask whether Theorem 4 can be generalized for the case where, in the two conditions of (9), one permits different γ values. The first such result was achieved by Heyde et al. [20]. ( see also Horvath [21]). To formulate their result, we need some notations and definitions.

Let

(12) $$n^{-1}\sum_{j=1}^{n}p_{j}^{-1}=2\gamma_{1}+\epsilon_{n},\;n^{-1}\sum_{j=-n}^{-1}p_{j}^{-1}=2\gamma_{2}+\epsilon_{n}^{*},$$

and define the function $\sigma^{2}\left(y\right)$ as

$$\sigma^{2}\left(y\right)=\left\{\begin{array}{cc} & \frac{1}{\gamma_{1}}\;\;\mathrm{for}\;y\geq 0,\\ & \frac{1}{\gamma_{2}}\;\;\mathrm{for}\;y<0. \end{array}\right.$$

Let {Y(t),t≥0} be a diffusion process on the same probability space as $\left\{C_{2}\left(N\right)\right\}$ defined by

$$Y\left(t\right)=W(A^{-1}\left(t\right)),\;t\geq 0,$$

where {W(t),t≥0} is a Wiener process,

$$A\left(t\right)=\int_{0}^{t}\sigma^{-2}\left(W\left(s\right)\right)\;ds,$$

and $A^{-1}\left(\cdot \right)$ is the inverse of A(·). If $\gamma_{1}\neq \gamma_{2}$, then Y(t) is a so-called oscillating Brownian motion, namely, a diffusion with the speed measure $m\left(dy\right)=2\sigma^{-2}\left(y\right)dy$ (see, e.g., Keilson and Wellner [22]).

Now, we are ready to formulate Heyde et al.’s result:

**Theorem** **6** ([20]). *Suppose that in* (12), $\epsilon_{k}$ and $\epsilon_{k}^{*}$ are o(1) as k→∞. Then, for $C_{2}\left(\cdot \right),$ we obtain the second coordinate of the anisotropic walk as N→∞:

$$\underset{0\leq t\leq N}{\sup}\left|N^{-1/2}C_{2}\left(\left[Nt\right]\right)-Y\left(t\right)\right|\to 0\;a.s.$$

Our aim was to have a general enough strong approximation for both coordinates with rates. This was partially achieved as follows.

**Theorem** **7** ([23]). *Suppose that*

(13) $$n^{-1}\sum_{j=1}^{n}p_{j}^{-1}=2\gamma_{1}+o\left(n^{-\eta }\right),\;n^{-1}\sum_{j=1}^{n}p_{-j}^{-1}=2\gamma_{2}+o\left(n^{-\eta }\right)$$

*as n→∞ for some constants $1<\max(\gamma_{1},\gamma_{2})<\infty$ and 1/2<η≤1. Under the conditions given in* (13)*, on an appropriate probability space for the anisotropic random walk $\left\{C\left(N\right)=\left(C_{1}\left(N\right),C_{2}\left(N\right)\right);\;\;N=0,1,2,\ldots \right\}$, one can construct two independent standard Wiener processes, $\left\{W_{1}\left(t\right);\;t\geq 0\right\}$ and $\left\{W_{2}\left(t\right);\;t\geq 0\right\}$, so that, as N→∞, we have, with any ε>0,*

(14) $$\left|C_{1}\left(N\right)-W_{1}\left(N-A_{2}^{-1}\left(N\right)\right)\right|+\left|C_{2}\left(N\right)-W_{2}\left(A_{2}^{-1}\left(N\right)\right)\right|=O\left(N^{5/8-\eta /4+\varepsilon }\right)\;a.s.,$$

*where $A_{2}\left(t\right)=\gamma_{1}\int_{0}^{t}I\left(W_{2}\left(s\right)\geq 0\right)\;ds+\gamma_{2}\int_{0}^{t}I\left(W_{2}\left(s\right)<0\right)\;ds$ and $A_{2}^{-1}\left(\cdot \right)$ is its inverse.*

**Remark** **2.** *We have to say that our goal was only partially achieved, as the $\gamma_{1}=\gamma_{2}=1$ case is not contained in the theorem.*

**Remark** **3.** *Observe that in the case that $\gamma_{1}=\gamma_{2}=\gamma >1$, $A\left(t\right)=\gamma t,\;\;A^{-1}\left(t\right)=t/\gamma$ and Y(t)=W(t/γ) is a time-changed Wiener process, and we recover Theorem 4.*

To shed some light on the extent of this result, we mention some important classes of anisotropic random walks.

- **Comb-Type walk:** We begin by defining an arbitrary subset B∈Z such that
  (15) $$p_{i}=1/4\;\mathrm{if}\;i\in B\;\mathrm{and}\;p_{i}=1/2\;\mathrm{if}\;i\in Z\setminus B.$$

So, in a comb-type walk, we remove from the two-dimensional integer lattice all the horizontal edges that do not belong to the *i*-levels in *B*, and in the remaining lattice, the walker takes each possible edge with equal probability.

In investigating this comb-type walk, we get rid of a lot of technicalities and still obtain interesting results.

- **Infinitely many horizontal lines:** Consider the case now where
  (16) $$|B_{n}|:=|B\cap \left\{-n,n\right\}|∼c\;n^{\beta }\;\mathrm{with}\;0<\beta \leq 1,\;c>0.$$

The β=1 case turns out to be a special case of Theorem 7; that is to say that under the conditions of Theorem 7, we obtain the same statement.

In order to be able to formulate our next result we need to say a few words about the method which we used here and in many of the other strong approximation results. Namely, we redefined the anisotropic walk in terms of two independent simple symmetric random walks, $S_{1}\left(\cdot \right)$ and $S_{2}\left(\cdot \right)$, and a sequence of independent geometric random variables $\left\{G_{i},i=1,2\ldots \right\}$ defined in the same probability space. Using these random variables, we define the anisotropic walk as taking $G_{i}$ horizontal steps and one vertical step for each i. The whole construction is given, e.g., in [24]. Now, we define

$$V_{N}=V_{N}\left(B\right)=\#\left\{k:\;1<k\leq N,\;\;\;C_{2}\left(k\right)\neq C_{2}\left(k-1\right)\right\},$$

which is the number of vertical steps, and denote by $H_{N}=N-V_{N}$ the number of horizontal steps in the first *N* steps. Then, as a result of the above-mentioned construction, we have

(17) $$\begin{array}{cc} & \left\{C(N);\;N=0,1,2,\ldots \right\}=\left\{(C_{1}\left(N\right),C_{2}\left(N\right));\;N=0,1,2,\ldots \right\}\\ \overset{d}{=} & \left\{(S_{1}\left(H_{N}\right),S_{2}\left(V_{N}\right));\;N=0,1,2,\ldots \right\}, \end{array}$$

where $\overset{d}{=}$ stands for equality in the distribution. Now, we are ready to formulate our next result. Let

$$\sum_{j\in B}\xi_{2}\left(j,V_{N}\right)$$

be the occupation time of *B* by $S_{2}\left(\cdot \right)$ in the first *N* steps of C(·), where $\left\{\xi_{2}\left(j,\cdot \right),\;j=0,\pm 1,\pm 2\ldots \right\}$ denotes the local time process of $S_{2}\left(\cdot \right).$

**Theorem** **8** ([24]). *Under the conditions* (15) *and* (16)*with 0<β<1, on an appropriate probability space for the comb-type walk $\left\{C\left(N\right)=\left(C_{1}\left(N\right),C_{2}\left(N\right)\right);N=0,1,2,\ldots \right\}$, one can construct two independent standard Wiener processes, $\left\{W_{1}\left(t\right);\;t\geq 0\right\}$ and $\left\{W_{2}\left(t\right);t\geq 0\right\}$ so that, as N→∞, we have, with any ε>0,*

$$\left|C_{1}\left(N\right)-W_{1}\left(\sum_{j\in B}\xi_{2}\left(j,V_{N}\right)\right)\right|=O\left(N^{1/8+\beta /8+\varepsilon }\right)\;a.s.$$

$$|C_{2}\left(N\right)-W_{2}\left(N\right)|=O\left(N^{1/4+\beta /4+\epsilon }\right)\;a.s.$$

It is not hard to check that with the notation $L_{2}\left(V_{N}\right):=\sum_{j\in B}\eta_{2}\left(j,V_{N}\right),$ where $\eta_{2}\left(j,\cdot \right)$ denotes the local time of $W_{2}\left(\cdot \right),$ we also have

$$|C_{1}\left(N\right)-W_{1}\left(L_{2}\left(V_{N}\right)\right)|=O\left(N^{1/8+\beta /4+\epsilon }\right)\;a.s.$$

Thus, we can replace the random walk local time with the corresponding Wiener local time. Furthermore, we can replace $V_{N}$ with *N* while obtaining a somewhat weaker rate:

$$|C_{1}\left(N\right)-W\left(L_{2}\left(N\right)\right)|=O\left(N^{1/8+3\beta /8+\epsilon }\right)\;a.s.$$

- **Half-plane, half-comb walk (HPHC)** is the case where we have B={0,1,2,…}; thus, under the *x*-axis, all horizontal lines are deleted. This model was discussed by us in [25,26]. In this case, Theorem 7 is true with $\gamma_{1}=2$ and $\gamma_{2}=1$ and the rate is somewhat better, as follows:

**Theorem** **9.** *On an appropriate probability space for the HPHC walk, one can construct two independent standard Wiener processes, $\left\{W_{1}\left(t\right);\;\;t\geq 0\right\}$ and $\{W_{2}\left(t\right);\;\;t\geq 0\},$ such that, as N→∞, we have, with any ϵ>0,*

$$\left|C_{1}\left(N\right)-W_{1}\left(N-A_{2}^{-1}\left(N\right)\right)\right|+\left|C_{2}\left(N\right)-W_{2}\left(A_{2}^{-1}\left(N\right)\right)\right|=O\left(N^{3/8+\varepsilon }\right)\;a.s.,$$

*with $A_{2}\left(t\right)=2\int_{0}^{t}I\left(W_{2}\left(s\right)\geq 0\right)\;ds+\int_{0}^{t}I\left(W_{2}\left(s\right)<0\right)\;ds.$*

- **Two-dimensional comb walk** (walk on $C^{2}$): This is one of the most important examples, where the set *B* only contains one element, B={0}; thus, we keep only the *x*-axis, and all the other horizontal lines are deleted. For this case, $\gamma_{1}=\gamma_{2}=1$, so Theorem 7 is not applicable. After Bertacchi’s remarkably weak convergence result [13], we proved the following.

**Theorem** **10** ([15]). *On an appropriate probability space for the comb walk* *$\left\{C\left(N\right)=\left(C_{1}\left(N\right),C_{2}\left(N\right)\right);\;\;N=0,1,2,\ldots \right\}$ on $C^{2}$, one can construct two independent standard Wiener processes, $\left\{W_{1}\left(t\right);\;\;t\geq 0\right\}$ and $\{W_{2}\left(t\right);\;\;t\geq 0\},$ such that, as N→∞, we have, with any ϵ>0,*

$$N^{-1/4}|C_{1}\left(N\right)-W_{1}\left(\eta_{2}\left(0,N\right)\right)|+N^{-1/2}\left|C_{2}\left(N\right)-W_{2}\left(N\right)\right|=O\left(N^{-1/8+\epsilon }\right)\;a.s.,$$

*where $\eta_{2}\left(0,\cdot \right)$ is the local time process at the zero of $W_{2}\left(\cdot \right).$*

- **K-comb walk** (walk on $C_{K}^{2}$): In this model, we relax the conditions of (15), replacing them with
  (18) $$p_{i}<1/2\;\mathrm{if}\;i\in B\;\mathrm{and}\;p_{i}=1/2\;\mathrm{if}\;i\in Z\setminus B,$$

but we require that |B| should be finite. In this case, by denoting |B|=K, the corresponding lattice by $C_{K}^{2}$ and introducing the notation

(19) $$A_{K}=\sum_{i\in B}\left(\frac{1}{2p_{i}}-1\right)$$

we proved the following theorem:

**Theorem** **11** ([27]). *On an appropriate probability space for the K-comb walk* *$\left\{C\left(N\right)=\left(C_{1}\left(N\right),C_{2}\left(N\right)\right);\;\;N=0,1,2,\ldots \right\}$ on $C_{K}^{2}$, one can construct two independent standard Wiener processes, $\left\{W_{1}\left(t\right);t\geq 0\right\}$ and $\{W_{2}\left(t\right);t\geq 0\},$ such that, as N→∞, we have, with any ϵ>0,*

$$N^{-1/4}|C_{1}\left(N\right)-W_{1}\left(A_{K}\eta_{2}\left(0,N\right)\right)|+N^{-1/2}\left|C_{2}\left(N\right)-W_{2}\left(N\right)\right|=O\left(N^{-1/8+\epsilon }\right)\;a.s.,$$

*where $\eta_{2}\left(0,\cdot \right)$ is the local time process at the zero of $W_{2}\left(\cdot \right).$*

**Remark** **4.** *Clearly, Theorem 11 is a generalization of Theorem 10. One of the interesting features of this result is that the positions of the K-horizontal lines are irrelevant.*

For the comb and the *K*-comb $\gamma_{1}=\gamma_{2}=1$, so they do not meet the conditions of Theorem 7, they need separate proofs. Our second open question is

**Question** **2.** *Find a generalization of Theorem 7 without the condition* $1<\max(\gamma_{1},\gamma_{2})<\infty .$ *It would be very nice to have only the condition of* $\max(\gamma_{1},\gamma_{2})<\infty$*, and the most ambitious version would be if we only needed to assume the recurrence of the anisotropic walk.*

## 4. Local Time

We spent a lot of time investigating this topic, and we proved many results for the different important special models, but there are many interesting questions which are still unanswered.

- **Periodic walk:** We managed to give a complete answer using some results from [7], which are known for this case only.

**Theorem** **12** ([19]). *For the 2N-step return probability of the periodic walk for any L≥1,*

(20) $$P\left(C^{P}\left(2N\right)=\left(0,0\right)\right)∼\frac{1}{4\pi Np_{0}\sqrt{\gamma -1}},\;as\;N\to \infty$$

*with γ given in* (11).

Observe that in the case where L=1 and $p_{0}=1/4$, γ=2 and we recover the well-known result for the simple symmetric two-dimensional walk. We remark that in the periodic case, γ is always bigger than 1.

From (20), we immediately obtain the truncated Green function

(21) $$g\left(N\right)=\sum_{k=0}^{N}P\left(C^{P}\left(k\right)=\left(0,0\right)\right)∼\frac{\log N}{4p_{0}\pi \sqrt{\gamma -1}},\;as\;N\to \infty .$$

Now, define the local time as

$$\Xi^{P}\left(\left(k,j\right),N\right)=:\sum_{r=1}^{N}I\left\{C^{P}\left(r\right)=\left(k,j\right)\right\},$$

where I{·} is the indicator function. Taking into account that our periodic walk is Harris-recurrent with the invariant measure given in (4), we obtain, using, e.g., Chen’s work [28], that

$$\lim_{N\to \infty }\frac{\Xi^{P}\left(\left(0,0\right),N\right)}{\Xi^{P}\left(\left(k,j\right),N\right)}=\frac{p_{j}}{p_{0}}\;a.s.$$

for fixed (k,j). Using g(N), we deduced many other results for the local time of the periodic walk, which we will mention later.

- **Two-dimensional comb walk:** In [16], we proved many results about local time on a generalized comb. Instead of introducing this general version, we want to recall here some of these results only in the context of the two-dimensional comb walk as it was given in [29].

**Theorem** **13.** *On the probability space of our Theorem 10, as N→∞, we have for the comb walk that*

$$\underset{x\in Z}{\sup}\left|\Xi \left(\left(x,0\right),N\right)-2\eta_{1}\left(x,\eta_{2}\left(0,N\right)\right)\right|=O\left(N^{1/8+\delta }\right)\;a.s.,$$

*with any δ>0, where $\eta_{i}\left(x,.\right),\;i=1,2$ are the local times of the corresponding independent Wiener processes in Theorem 10. Furthermore, we have, for any 0<ε<1/4,*

(22) $$\max_{\left|x\right|\leq N^{1/4-\varepsilon }}\left|\Xi \left(\left(x,0\right),N\right)-\Xi \left(\left(0,0\right),N\right)\right|=O\left(N^{1/4-\delta }\right)\;a.s.$$

*and*

$$\begin{array}{c} \max_{0<|y|\leq N^{1/4-\varepsilon }}\max_{\left|x\right|\leq N^{1/4-\varepsilon }}\left|\Xi \left(\left(x,y\right),N\right)-\frac{1}{2}\Xi \left(\left(0,0\right),N\right)\right|=O\left(N^{1/4-\delta }\right)\;a.s., \end{array}$$

*for any 0<δ<ε/2, where the maximum is taken at integers.*

Here, the first statement of the theorem describes the behavior of the local time of the sites on the *x*-axis. The second statement claims that on the *x*-axis, up to $\left|x\right|\leq N^{1/4-\varepsilon }$, all sites have approximately the same local time, while the last statement explains that away from the *x*-axis, the local time is approximately half of the local time at the origin. Theorem 13 has many nice consequences, which are given in [16]. These results were obtained using strong approximation methods. However, we did not calculate the 2N-step return probability to the origin. This was achieved much earlier by Gerl [30]. He even proved it for higher-dimensional combs. He obtained the following result:

**Theorem** **14** ([30]). *For the two-dimensional comb walk, as N→∞,*

(23) $$P\left(C\left(2N\right)=\left(0,0\right)\right)∼\frac{1}{\sqrt{2}\;\Gamma \left(1/4\right)N^{3/4}}.$$

His proof strongly relies on the fact that the comb lattice is a tree. So, here is our next question:

**Question** **3.** *Find a probabilistic proof of (23) and generalize it for the K-comb.*

- **Periodic-like walk:**

In our paper, we proved the following:

**Theorem** **15** ([31])**.** *Consider the anisotropic walk defined in* (1)*. Suppose that*

(i) *Conditions (9) hold with some 1<γ<∞ and 1/2<η<∞.*
(ii) *There is an ω>0 such that $p_{j}\geq \omega ,\;j=0,\pm 1,\pm 2,\ldots$*
(iii) $\sup_{n\geq 0}\sqrt{n}\sup_{N\geq n}\sum_{m=n}^{N}\left(P\left(C_{2}\left(2m+2\right)=0\right)-P\left(C_{2}\left(2m+1\right)=0\right)\right)<\infty .$

*Then,*

(24) $$P\left(C\left(2N\right)=\left(0,0\right)\right)∼\frac{1}{4Np_{0}\pi \sqrt{\gamma -1}},\;\mathrm{as}\;N\to \infty .$$

It is very interesting that the limiting 2N-step return probability in this theorem is identical to the result that we obtained in the case of the periodic walk. This is the only reason that we call the anisotropic walks which satisfy the three conditions listed in Theorem 15 **periodic-like**.

As before, this theorem immediately implies that the truncated Green function is

$$g\left(N\right)=\sum_{k=0}^{N}P\left(C\left(k\right)=\left(0,0\right)\right)∼\frac{\log N}{4p_{0}\pi \sqrt{\gamma -1}},\;as\;N\to \infty ,$$

from which, using Chen’s work [28] again, we can conclude that for any fixed (x,y),

$$\lim_{N\to \infty }\frac{\Xi ((0,0),N)}{\Xi ((x,y),N)}=\frac{p_{y}}{p_{0}}\;a.s.$$

In the proof of the above theorem, we used a local limit theorem for a certain type of Markov chain introduced by Stenlund [32]. The Markov chain he deals with goes from state *i* to states i+1 and i−1 with the same probability $p_{i}$ and remains in state *i* with a probability of $1-2p_{i}.$ This is exactly what the second coordinate, $C_{2}\left(\cdot \right)$, of our anisotropic walk does.

**Remark** **5.** *In Theorem 15, condition* (iii) *is difficult to check. This condition comes from Stenlund’s paper, where he made the following comment on this: if the walk is lazy, i.e., all $p_{j}\leq 1/4,$ then* (iii) *is trivially satisfied. His simulation suggests that, generally, under the other conditions of Theorem 15, condition* (iii) *holds if $p_{k}\neq 1/2$ for at least one k∈Z. So* (iii) *could well turn out to be equivalent to the Markov chain being aperiodic.*

**Question** **4.** *Find a class of anisotropic random walks for which condition (iii) above holds (besides being lazy).*

**Question** **5.** *Find some conditions which are equivalent to condition (iii) but simpler to check*.

This theorem brings up the most puzzling questions. In Theorems 12 and 15, we obtained identical results. The first one was for the periodic walk (see the definition of periodic walk in Section 3, which is very different from periodic Markov chains), and the second was for periodic-like walks, that is, under the three conditions listed above. Clearly, the periodic walk satisfies (i) and (ii) above. So, either every periodic walk satisfies condition (iii) in Theorem 15, which we cannot prove or disprove, or there is a common generalization of Theorems 12 and 15.

**Question** **6.** *Find a common generalization of Theorems 12 and 15.*

Clearly, (24) does not make sense when γ=1. So, we would like to have a result which contains the K-comb given in (24) and, if possible, all anisotropic walks with γ=1.

The only case where we managed to find local time results for the anisotropic walk, when $\gamma_{1}\neq \gamma_{2}$, was the following:

- **The half-plane, half-comb walk.**

**Theorem** **16** ([26]). *For the 2N-step return probability of the HPHC walk, starting at (0,0), we have*

(25) $$P\left(C\left(2N\right)=\left(0,0\right)\right)∼\frac{2}{\pi N},\;as\;N\to \infty .$$

Of course, this result allows us to calculate the truncated Green function and proceed similarly to the way we described above in the case of the periodic walk. In this particular case, $\gamma_{1}=2$ and $\gamma_{2}=1.$

**Question** **7.** *What is the reason that the asymptotic return probability of the HPHC walk is twice as much as that of the simple symmetric walk on the plane? Even a heuristic explanation would be nice.*

**Question** **8.** *Find a general result for the asymptotic 2N-step return probability in the case of* $\gamma_{1}=\gamma_{2},$ *under weaker conditions than the periodicity.*

**Question** **9.** *Find a general result for the asymptotic 2N-step return probability in the case of* $\gamma_{1}\neq \gamma_{2}.$

## 5. Range

The range is defined as the number of sites visited by the walk during the first *N* steps. Formally, it is defined as

$$R\left(N\right)=\sum_{u\in Z^{2}}I\left\{\Xi \left(u,N\right)>0\right\},$$

where I{·} is the indicator function. In the case of the simple symmetric walk in two dimensions, we know from Dvoretzky and Erdős [10] that

$$E\left(R\left(N\right)\right)=\frac{\pi N}{\log N}+O\left(\frac{N\log\log N}{{\left(\log N\right)}^{2}}\right)$$

and that the following law of large numbers holds:

$$\lim_{N\to \infty }\frac{R(N)}{E(R(N))}=1\;a.s.$$

As for the anisotropic walk, we know very little.

- **Periodic walk**

Roerdink and Shuler [7] proved that

$$E\left(R\left(N\right)\right)∼\frac{2\pi \sqrt{\gamma -1}}{\gamma }\frac{N}{\log N},\;as\;N\to \infty .$$

Furthermore, from Nándori’s work [33], one can conclude the following laws of large numbers:

$$\lim_{N\to \infty }\frac{R(N)}{E(R(N))}=\lim_{N\to \infty }\frac{\gamma \;R(N)\log N}{2\pi \sqrt{\gamma -1}\;N}=1\;a.s.$$

**Question** **10.** *How to generalize these results for the anisotropic walk, without the restriction of periodicity, requesting only that* γ>1.

- **Two-dimensional comb walk**

Pach and Tardos [34] proved for the expected value of the range of the comb that

$$E\left(R\left(N\right)\right)=\left(\frac{1}{2\sqrt{2\pi }}+o\left(1\right)\right)\sqrt{N}\log N,\;\mathrm{as}\;N\to \infty .$$

As to the almost sure behavior of the range of the comb walk, we just have some crude estimates. We can create a lower bound by observing that the range of the vertical walk, $C_{2}\left(\cdot \right)$, is the lower bound of the range of the comb. We proved in [15] that

$$\underset{n\to \infty }{lim inf}{\left(\frac{8\log\log n}{\pi^{2}n}\right)}^{1/2}\max_{1\leq k\leq n}\left|C_{2}\left(k\right)\right|=1\;a.s.,$$

so

$$\underset{n\to \infty }{lim inf}\frac{\sqrt{8\log\log n}}{\pi \sqrt{n}}R_{n}\geq 1\;a.s.$$

In order to obtain an upper bound for the range of the comb, we prove that

(26) $$\underset{n\to \infty }{lim sup}\frac{R_{n}}{n^{1/2}\log n{\left(\log\log n\right)}^{5/2+\epsilon }}\leq c\;a.s.,$$

with some c>0. To see this, first, we recall a few facts.

We proved in [15] that

(27) $$\;\underset{n\to \infty }{lim sup}\frac{C_{1}\left(n\right)}{n^{1/4}{\left(\log\log n\right)}^{3/4}}=\frac{2^{5/4}}{3^{3/4}}\;a.s.,$$

which means that if *n* is big enough, then in *n* steps, we do not have more than

const$n^{1/4}{\left(\log\log n\right)}^{3/4}$ sites occupied on the *x*-axis. From (34) (see later), we also know that from none of these sites can we have more than $const\;n^{1/4}{\left(\log\log n\right)}^{3/4}$ excursions if *n* is big enough.

If κ is an excursion away from the *x*-axis, then for its height *H*, we have that P(H≥k)=1/k. So, we fix a site (x,0) on the *x*-axis. If {$\kappa_{i}\;\;i=1,2,\ldots \}$ is the *i*-th excursion away from (x,0) and its height is $\left\{H_{i}\;\;i=1,2,\ldots \right\}$, then for the probability that if in the first *n* step $m_{n}$ excursions start from (x,0), at least one of them is higher than $\ell_{n}$, we have

$$P\left(\max_{i\leq m_{n}}\kappa_{i}>\ell_{n}\right)=1-{\left(1-\frac{1}{\ell_{n}}\right)}^{m_{n}}\leq 1-e^{-\frac{m_{n}}{\ell_{n}}}\leq \frac{m_{n}}{\ell_{n}}.$$

Select $m_{n}=\alpha n^{1/4}{\left(\log\log n\right)}^{3/4}\;\mathrm{and}\;\ell_{n}=\beta n^{1/4}\log n{\left(\log\log n\right)}^{7/4+\epsilon }$ with some α>0 and β>0. Let $n_{k}=e^{k}.$ Then,

(28) $$\begin{array}{ccc} & & P\left(\max_{i\leq m_{n_{k+1}}}\kappa_{i}>\ell_{n_{k}}\right)\leq \frac{m_{n_{k+1}}}{\ell_{n_{k}}}=\frac{\alpha }{\beta }{\left(\frac{n_{k+1}}{n_{k}}\right)}^{1/4}{\left(\frac{\log\log n_{k+1}}{\log\log n_{k}}\right)}^{3/4}\frac{1}{k{\left(\log k\right)}^{1+\epsilon }}\\ & \leq & const\;{\left(\frac{\log(k+1)}{\log k}\right)}^{3/4}\frac{1}{k{\left(\log k\right)}^{1+\epsilon }}. \end{array}$$

So,

$$\sum_{k}P\left(\max_{i\leq m_{n_{k+1}}}\kappa_{i}>\ell_{n_{k}}\right)$$

is finite. Hence, for a big enough *k*,

$$\max_{i\leq m_{n_{k+1}}}\kappa_{i}\leq \ell_{n_{k}}.$$

So, for $n_{k}\leq n\leq n_{k+1}$, we have

$$\max_{i\leq m_{n}}\kappa_{i}\leq \max_{i\leq m_{n_{k+1}}}\kappa_{i}\leq \ell_{n_{k}}\leq \ell_{n}\;a.s.$$

if *k* is big enough. Thus, in *n* steps in each tooth, we have, at most, $\beta n^{1/4}\log n{\left(\log\log n\right)}^{7/4+\epsilon }$ points visited. Now, using the fact that according to (27) at most $const\;n^{1/4}{\left(\log\log n\right)}^{3/4}$ sites are visited on the *x*-axis, we obtain

$$R_{n}\leq const\;n^{1/4}{\left(\log\log n\right)}^{3/4})\beta n^{1/4}\log n{\left(\log\log n\right)}^{7/4+\epsilon }=const\;n^{1/2}\log n{\left(\log\log n\right)}^{5/2+\epsilon },$$

proving (26).

**Question** **11.** *Find some exact result about the range of the comb walk.*

**Question** **12.** *What is the range of the K-comb walk and the HPHC walk.*

## 6. Strassen Theorems and Strong Laws for the Coordinates of the Anisotropic Walks

In this section, we want to mention some of the consequences of the strong approximation results discussed in Section 3. In order to mention our Strassen theorems, we need some definitions.

Let S be the Strassen class of functions, i.e., S⊂C([0,1],R) is the class of absolutely continuous functions (with respect to the Lebesgue measure) on [0,1] for which

(29) $$f\left(0\right)=0\;\mathrm{and}\;\int_{0}^{1}{\dot{f}}^{2}\left(x\right)dx\leq 1.$$

**Remark** **6.** *In this topic, instead of the usual $f^{\prime }\left(\cdot \right)$ for the derivative, we use the traditional $\dot{f}\left(\cdot \right)$ notation; see, e.g.,* [35].

Define the Strassen class $S^{2}$ as the set of $R^{2}$-valued, absolutely continuous functions

(30) {(f(x),g(x));0≤x≤1}

for which f(0)=g(0)=0 and

(31) $$\int_{0}^{1}\left({\dot{f}}^{2}\left(x\right)+{\dot{g}}^{2}\left(x\right)\right)dx\leq 1.$$

At first, we mention the general case

**Theorem 17**  ([19])**.** *Under the conditions of Theorem 4 for the anisotropic walk C(·), we have that the sequence of random vector-valued functions*

$${\left(\sqrt{\frac{\gamma }{\gamma -1}}\frac{C_{1}\left(xN\right)}{{\left(2N\log\log N\right)}^{1/2}},\sqrt{\gamma }\frac{C_{2}\left(xN\right)}{{\left(2N\log\log N\right)}^{1/2}},\;\;\;\;0\leq x\leq 1\right)}_{N\geq 3}$$

*is almost surely relatively compact in the space $C(\left[0,1\right],R^{2})$ and its limit points are the set of functions $S^{2}.$ In particular, the vector sequence*

$${\left(\frac{C_{1}\left(N\right)}{{\left(2N\log\log N\right)}^{1/2}},\frac{C_{2}\left(N\right)}{{\left(2N\log\log N\right)}^{1/2}}\right)}_{N\geq 3}$$

*is almost surely relatively compact in the rectangle*

$$\left[-\frac{\sqrt{\gamma -1}}{\sqrt{\gamma }},\frac{\sqrt{\gamma -1}}{\sqrt{\gamma }}\right]\times \left[-\frac{1}{\sqrt{\gamma }},\frac{1}{\sqrt{\gamma }}\right],$$

*and the set of its limit points is the ellipse*

(32) $$\left\{\left(x,y\right):\frac{\gamma }{\gamma -1}x^{2}+\gamma y^{2}\leq 1\right\}.$$

We do not have any Strassen-type results if $\gamma_{1}\neq \gamma_{2},$ so we ask the following:

**Question** **13.** *How can we generalize Theorem 17 for the case where* $\gamma_{1}\neq \gamma_{2}.$

The only other case where we managed to prove a Strassen theorem was the two-dimensional comb walk.

**Theorem** **18** ([15]). *For the random walk $\left\{C\left(n\right)=\left(C_{1}\left(n\right),C_{2}\left(n\right)\right);\;n=1,2,\ldots \right\}$ on the* two *-dimensional comb lattice $C^{2}$, we have that the sequence of random vector-valued functions*

$$\begin{array}{c} {\left(\frac{C_{1}\left(xn\right)}{2^{3/4}n^{1/4}{\left(\log\log n\right)}^{3/4}},\frac{C_{2}\left(xn\right)}{{\left(2n\log\log n\right)}^{1/2}},\;0\leq x\leq 1\right)}_{n\geq 3} \end{array}$$

*is almost surely compact in the space $C(\left[0,1\right],R^{2})$, and its limit points are the set of functions*

(33) $$\begin{array}{ccc} S^{\left(2\right)}: & = & \left\{\left(k\left(x\right),g\left(x\right)\right):\;k\left(0\right)=g\left(0\right)=0,\;k,g\in \dot{C}\left(\left[0,1\right],R\right)\right\}\\ & & \int_{0}^{1}\left(\right|3^{3/4}2^{-1/2}\dot{k}\left(x\right){|}^{4/3}+{\dot{g}}^{2}\left(x\right))\;dx\leq 1,\;\dot{k}\left(x\right)g\left(x\right)=0\;\;a.e., \end{array}$$

*where $\dot{C}\left(\left[0,1\right],R\right)$ stands for the space of absolutely continuous functions in C([0,1],R).*

*The sequence*

$${\left(\frac{C_{1}\left(n\right)}{n^{1/4}{\left(\log\log n\right)}^{3/4}},\frac{C_{2}\left(n\right)}{{\left(2n\log\log n\right)}^{1/2}}\right)}_{n\geq 3}$$

*is almost surely compact in the rectangle*

$$R=\left[-\frac{2^{5/4}}{3^{3/4}},\frac{2^{5/4}}{3^{3/4}}\right]\times \left[-1,1\right],$$

*and the set of its limit points is the domain*

$$D=\{\left(u,v\right):\;k\left(1\right)=u,\;g\left(1\right)=v,\;\left(k\left(\cdot \right),g\left(\cdot \right)\right)\in S^{\left(2\right)}\}.$$

In fact, in [15], we established some other important consequences of this theorem as well.

**Question** **14.** * How to generalize Theorem 18 for the K-comb walk.*

In what follows, we present some LIL-type theorems.

**Theorem** **19** ([23]). *Under the conditions of Theorem 7, we have the following LIL-type results for the anisotropic walk:*

$$\begin{array}{ccc} & • & \underset{t\to \infty }{lim sup}\frac{W_{1}\left(t-A_{2}^{-1}\left(t\right)\right)}{\sqrt{t\log\log t}}=\underset{N\to \infty }{lim sup}\frac{C_{1}\left(N\right)}{\sqrt{N\log\log N}}=\sqrt{2\left(1-\frac{1}{\gamma_{1}}\right)}\;a.s.;\\ & • & \underset{t\to \infty }{lim inf}\frac{W_{1}\left(t-A_{2}^{-1}\left(t\right)\right)}{\sqrt{t\log\log t}}=\underset{N\to \infty }{lim inf}\frac{C_{1}\left(N\right)}{\sqrt{N\log\log N}}=-\sqrt{2\left(1-\frac{1}{\gamma_{1}}\right)}\;a.s.;\\ & • & \underset{t\to \infty }{lim sup}\frac{W_{2}\left(A_{2}^{-1}\left(t\right)\right)}{\sqrt{t\log\log t}}=\underset{N\to \infty }{lim sup}\frac{C_{2}\left(N\right)}{\sqrt{N\log\log N}}=\sqrt{\frac{2}{\gamma_{1}}}\;a.s.;\\ & • & \underset{t\to \infty }{lim inf}\frac{W_{2}\left(A_{2}^{-1}\left(t\right)\right)}{\sqrt{t\log\log t}}=\underset{N\to \infty }{lim inf}\frac{C_{2}\left(N\right)}{\sqrt{N\log\log N}}=-\sqrt{\frac{2}{\gamma_{2}}}\;a.s. \end{array}$$

*where $A_{2}\left(t\right)=\gamma_{1}\int_{0}^{t}I\left(W_{2}\left(s\right)\geq 0\right)\;ds+\gamma_{2}\int_{0}^{t}I\left(W_{2}\left(s\right)<0\right)\;ds,$ and $A_{2}^{-1}\left(\cdot \right)$ is its inverse.*

This theorem contains almost all the special cases which we discussed earlier. For instance, in the **HPHC walk** case, where we have $\gamma_{1}=2$ and $\gamma_{2}=1$, we obtain

$$\begin{array}{ccc} & • & \underset{N\to \infty }{lim sup}\frac{C_{1}\left(N\right)}{\sqrt{N\log\log N}}=1\;a.s.;\\ & • & \underset{N\to \infty }{lim inf}\frac{C_{1}\left(N\right)}{\sqrt{N\log\log N}}=-1\;a.s.;\\ & • & \underset{N\to \infty }{lim sup}\frac{C_{2}\left(N\right)}{\sqrt{N\log\log N}}=1\;a.s.;\\ & • & \underset{N\to \infty }{lim inf}\frac{C_{2}\left(N\right)}{\sqrt{N\log\log N}}=-\sqrt{2}\;a.s. \end{array}$$

However, again, the $\gamma_{1}=\gamma_{2}=1$ case in general is unknown, but we have some LIL for the **K-comb walk**.

**Theorem** **20** ([27]). *For the K-comb walk, we have*

$$•\;\underset{N\to \infty }{lim sup}\frac{C_{1}\left(N\right)}{\sqrt{A_{K}}N^{1/4}{\left(\log\log N\right)}^{3/4}}=\frac{2^{5/4}}{3^{3/4}}\;a.s.,$$

$$•\;\underset{N\to \infty }{lim sup}\frac{C_{2}\left(N\right)}{{\left(2N\log\log N\right)}^{1/2}}=1\;a.s.,\;$$

*where $A_{K}$ was defined in* (19).

*Furthermore, let ρ(n),n=1,2…, be a non-increasing sequence of positive numbers such that $n^{1/4}\rho \left(n\right)$ is non-decreasing. Then, we have almost surely that*

$$•\;\underset{N\to \infty }{lim inf}\frac{max_{0\leq k\leq N}C_{1}\left(k\right)}{N^{1/4}\rho \left(N\right)}=0\;\;or\;\;\infty$$

$$•\;\underset{N\to \infty }{lim inf}\frac{max_{0\leq k\leq N}C_{2}\left(k\right)}{N^{1/2}\rho \left(N\right)}=0\;\;or\;\;\infty$$

*according to whether the series $\sum_{i=1}^{\infty }\rho \left(n\right)/n$ diverges or converges.*

*Furthermore, for $|C_{2}\left(\cdot \right)|$ and $|C_{1}\left(\cdot \right)|$, we have*

$$•\;\;\underset{N\to \infty }{lim inf}{\left(\frac{8\log\log N}{\pi^{2}N}\right)}^{1/2}\max_{0\leq k\leq N}\left|C_{2}\left(k\right)\right|=1\;a.s.$$

$$•\;\underset{N\to \infty }{lim inf}\frac{\max_{0\leq k\leq N}\left|C_{1}\left(k\right)\right|}{N^{1/4}\rho \left(N\right)}=0\;or\;\infty ,\;a.s.$$

*as to whether the series $\sum_{n=1}^{\infty }\rho^{2}\left(n\right)/n$ diverges or converges.*

## 7. Some Limit Distributions and Strong Laws for the Local Time of the Anisotropic Walk

- **Periodic and periodic-like walk**

From (11), using the work of Darling and Kac [36], we concluded in [19] that for the periodic walk,

$$•\;\lim_{N\to \infty }P\left(\frac{4p_{0}\pi \sqrt{\gamma -1}\;\Xi \left(\left(0,0\right),n\right)}{\log n}\geq x\right)=e^{-x},\;x\geq 0.$$

As for the lim sup result, we have, using Chen’s work [28], that

$$•\;\underset{n\to \infty }{lim sup}\frac{\Xi ((0,0),n)}{\log n\log\log\log n}=\frac{1}{4p_{0}\pi \sqrt{\gamma -1}}\;a.s.$$

Naturally, the above two statements are also valid for the periodic-like walk as well. A functional limit theorem was also proved in [37].

- **Two-dimensional comb walk**

Based on an old Strassen theorem [38], which we proved for the iterated Wiener local time process $\left\{\eta_{1}\left(0,\eta_{2}\left(0,xt\right)\right),\;\;0\leq x\leq 1\right\}$, where $\eta_{1}\left(\cdot \right)$ and $\eta_{2}\left(\cdot \right)$ are the local times of two independent standard Wiener processes, we concluded in [16] that for the local time of the comb, we have

(34) $$•\;\underset{n\to \infty }{lim sup}\frac{\Xi ((x,0),n)}{n^{1/4}{\left(\log\log n\right)}^{3/4}}=\frac{2^{9/4}}{3^{3/4}}\;a.s.,$$

$$•\;\underset{n\to \infty }{lim sup}\frac{\Xi ((x,y),n)}{n^{1/4}{\left(\log\log n\right)}^{3/4}}=\frac{2^{5/4}}{3^{3/4}}\;y\neq 0\;\;a.s.$$

Furthermore, we would like to mention a couple of integral tests for $\sup_{x\in Z}\Xi \left(\left(x,0\right),n\right)$ [17].

**Theorem** **21** ([17]). *Let a(n) be a non-decreasing sequence. Then, as n→∞,*

$$P(\underset{x\in Z}{\sup}\Xi \left(\left(x,0\right),n\right)>n^{1/4}a\left(n\right)\;\;i.o.)=0\;\;\;or\;\;\;1$$

*according to*

$$\sum_{n=1}^{\infty }\frac{a^{2}\left(n\right)}{n}\exp\left(-\frac{3a^{4/3}\left(n\right)}{2^{5/3}}\right)<\infty \;\;\;or\;\;\;=\infty .$$

**Theorem** **22** ([17]). *Let b(n) be a non-increasing sequence. Then, as n→∞,*

$$P(\underset{x\in Z}{\sup}\Xi \left(\left(x,0\right),n\right)<n^{1/4}b\left(n\right)\;\;\;i.o.)=0\;\;\;or\;\;\;1$$

*according to*

$$\sum_{n=1}^{\infty }\frac{b^{2}\left(n\right)}{n}<\infty \;\;\;or\;\;\;=\infty .$$

- **HPHC walk**

As a consequence of Theorem 16, we have for the HPHC walk that $g\left(N\right)∼\frac{2}{\pi }\log N.$ In [26], we obtained again from Darling and Kac [36] and Chen [28] that

$$•\;\lim_{N\to \infty }P\left(\frac{\pi \;\Xi ((0,0),N)}{2\log N}\geq x\right)=e^{-x}.$$

and

$$•\;\underset{N\to \infty }{lim sup}\frac{\Xi ((0,0),N)}{\log N\log\log\log N}=\frac{2}{\pi }\;a.s.$$

## Data Availability

No new data were created or analyzed in this study.
